# Bacterial and fungal communities and network dynamics in Otitis media patients upon 1,8-Cineol treatment

**DOI:** 10.1038/s41540-026-00808-x

**Published:** 2026-08-11

**Authors:** Anke Leichtle, Axel Künstner, Zuzana Penxová, Simon Graspeuntner, Jonas Fleckner, Sven Künzel, Daniel Drömann, Hauke Busch, Ralph Pries, Karl-Ludwig Bruchhage

**Affiliations:** 1 Department of Otorhinolaryngology, University of Luebeck, Luebeck, Germany; 2 Department of Otorhinolaryngology, Hannover Medical School, Hannover, Germany; 3 Luebeck Institute of Experimental Dermatology (LIED), University of Luebeck, Medical Systems Biology Group, Luebeck, Germany; 4 Medical Clinic III, University of Luebeck, Luebeck, Germany; 5 Max-Planck Institute for Evolutionary Biology, Plön, Germany

**Keywords:** Diseases, Microbiology

## Abstract

The present study investigated the bacterial and fungal communities and cross-domain network interactions in patients with chronic Otitis media in response to the natural monoterpene medicinal 1,8-Cineol as a promising treatment option. While the bacterial community showed modest changes with preserved core genera, the fungal community underwent dramatic diversification and network expansion. This suggests that treatment may have disrupted competitive exclusion mechanisms in the latter, allowing for increased diversity and interactions, despite no significant changes in traditional diversity metrics. Investigations on the microbial community compositions in Otitis media patients upon 1,8-Cineol treatment revealed significantly different initial microbial signatures in responders and non-responders. Notably, therapy-responding COM patients demonstrated a baseline situation with significantly higher abundances of different gram-negative *Pseudomonas* species. In contrast, significantly elevated levels of various gram-positive *Corynebacterium* species were observed in the non-responder group compared to the 1,8-Cineol responding patients. These keystone taxa may represent new targets for therapeutic intervention or biomarkers for treatment response.

## Introduction

Chronic otitis media (COM) is a persistent inflammation of the middle ear and is among the most prevalent ear diseases worldwide, associated with substantial healthcare costs, significant impairments in patients‘ quality of life, and represents one of the leading causes of acquired and preventable hearing loss^1,2^. The clinical picture of this widespread pathology includes ear discharge and hearing loss, and often irreversible destruction of the tympanic membrane and the surrounding middle ear structures if timely treatment is not provided^3–5^.

The pathogens primarily responsible for COM development are *Pseudomonas aeruginosa, Proteus mirabilis*, *Staphylococcus aureus*, and *Enterobacterales*, *Candida* species, and different anaerobic bacteria, all of which are also associated with advanced disease processes such as mastoiditis^6,7^.

Besides surgery, the standard treatment for persistent COM is the systemic administration of antibiotics, including penicillin, cephalosporins, and gyrase inhibitors, to reduce the bacterial infection^8^. In this context, potential side effects in response to antibiotic administration have to be considered, since moderate and high levels of multidrug-resistance, especially to 3rd generation cephalosporins, are increasingly observed^9^.

Furthermore, topical therapy with aminoglycosides, such as neomycin, has been shown to induce cytotoxicity in the cochlea and the vestibular system and, therefore, should be administered with careful consideration, especially to children, as the most vulnerable part of the population^10–12^. Hence, an evaluation of alternative treatment options for COM patients is required to deal with therapy resistance and undesirable side effects of antibiotic administration.

In this context, the natural anti-inflammatory monoterpene 1,8-Cineol is a very promising option. 1,8-Cineol is commonly used as the clinically approved drug Soledum^®^ for the treatment of different chronic and acute airway diseases and has been shown to significantly reduce leukocyte-derived pro-inflammatory cytokines such as TNF-α, IL-1β, IL-4, or IL-6^13,14^. Recent investigations indicated a systemic distribution of orally administered 1,8-Cineol in the human body via the gut and the bloodstream, from where it is expelled through the lungs and the respiratory tract along the mucosal tissues^15^.

Off-label treatment of antibiotic-resistant COM patients with 1,8-Cineol revealed significantly reduced levels of inflammatory pathogens in ear swab samples. While gut microbiota appear to be stable throughout 1,8-Cineol treatment^16^. An association of intestinal *Prevotella copri* with an improved clinical outcome in specific individuals has been observed^17^. However, there is currently no data regarding the influence of 1,8-Cineol treatment on the dynamics of bacterial and fungal communities and their interactions in COM patients.

In view of inflammatory diseases, investigations have thus far primarily focused on interactions between patients and disease-associated bacteria. In contrast, cross-domain interactions between bacteria and fungi have only very rarely been studied^18^.

Therefore, in this study, we investigated ear swabs of COM patients before and after 14 days of oral administration of 1,8-Cineol (CNL-1976^®^) for DNA isolation and sequencing analysis of the existing bacterial and fungal communities of the ear. Comprehensive network analyses were performed using a correlation-based approach to identify significant co-occurrence patterns within the bacterial and fungal domains and to characterize underlying network properties. Data were correlated regarding therapy response and the clinical situation of our patients to improve the current treatment options and possible forms of application of this natural drug.

## Results

### Bacterial and fungal communities in response to 1,8-Cineol

Swabs were taken from the auditory canal of COM patients before and after 14 days of oral administration of 1,8-Cineol capsules. Microbiological DNA sequencing analyses were carried out, whereas species occurring in less than 10% of the samples, as well as samples with <500 counts (bacteria) or <200 counts (fungi), were removed.

**Fig. 1:**
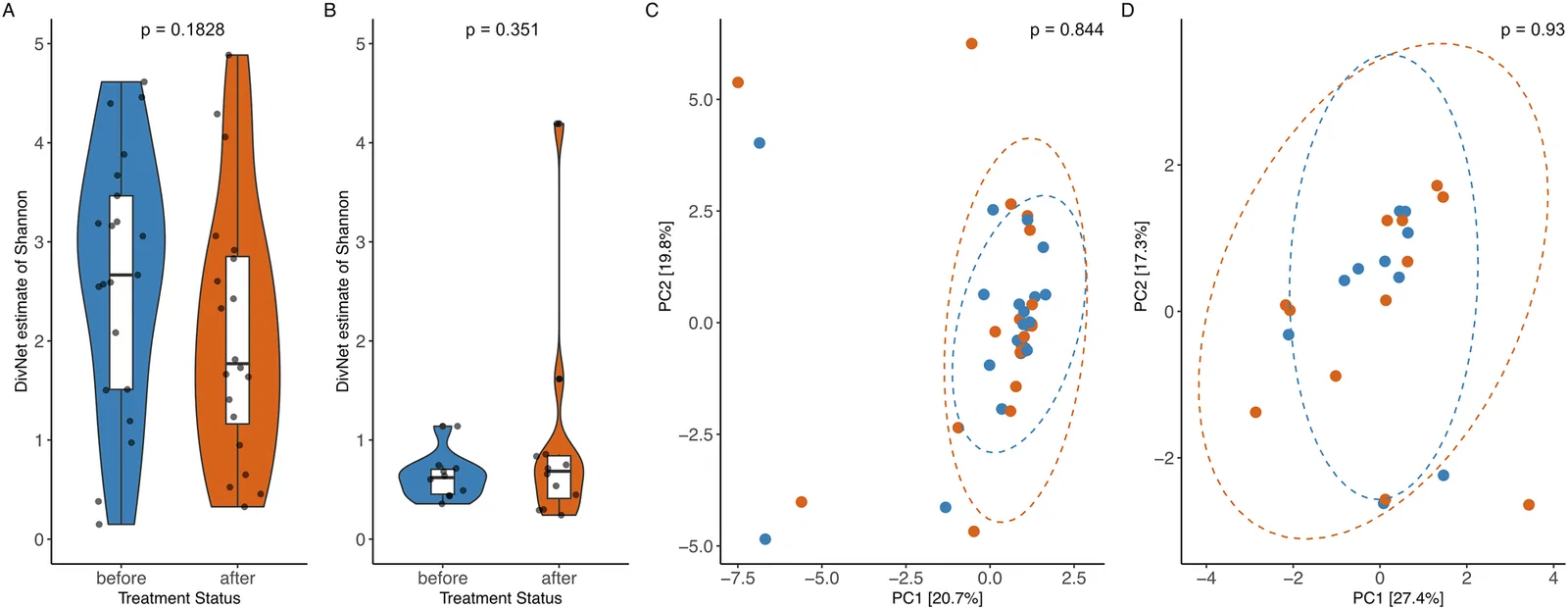
Analyses of alpha and beta diversities revealed no significant differences in bacterial or fungal communities upon 1,8-Cineol treatment. Alpha diversity of bacteria is shown in (**A**) and fungal alpha diversity in (**B**) with *p* values derived from the betta test. Principal component analysis (PCA) graph (**C**) shows beta diversity in bacteria and **D** in fungi; *p* values derived from PERMANOVA. Blue denotes samples before treatment, orange after treatment, respectively.

No significant differences were detected in alpha diversity (Shannon index) for either bacteria (*p* = 0.092) or fungi (*p* = 0.166). Moreover, beta diversity (Aitchison distance) showed no significant community-level shifts (PERMANOVA *p* = 0.844 for bacteria, *p* = 0.93 for fungi) (Fig. 1).

**Fig. 2:**
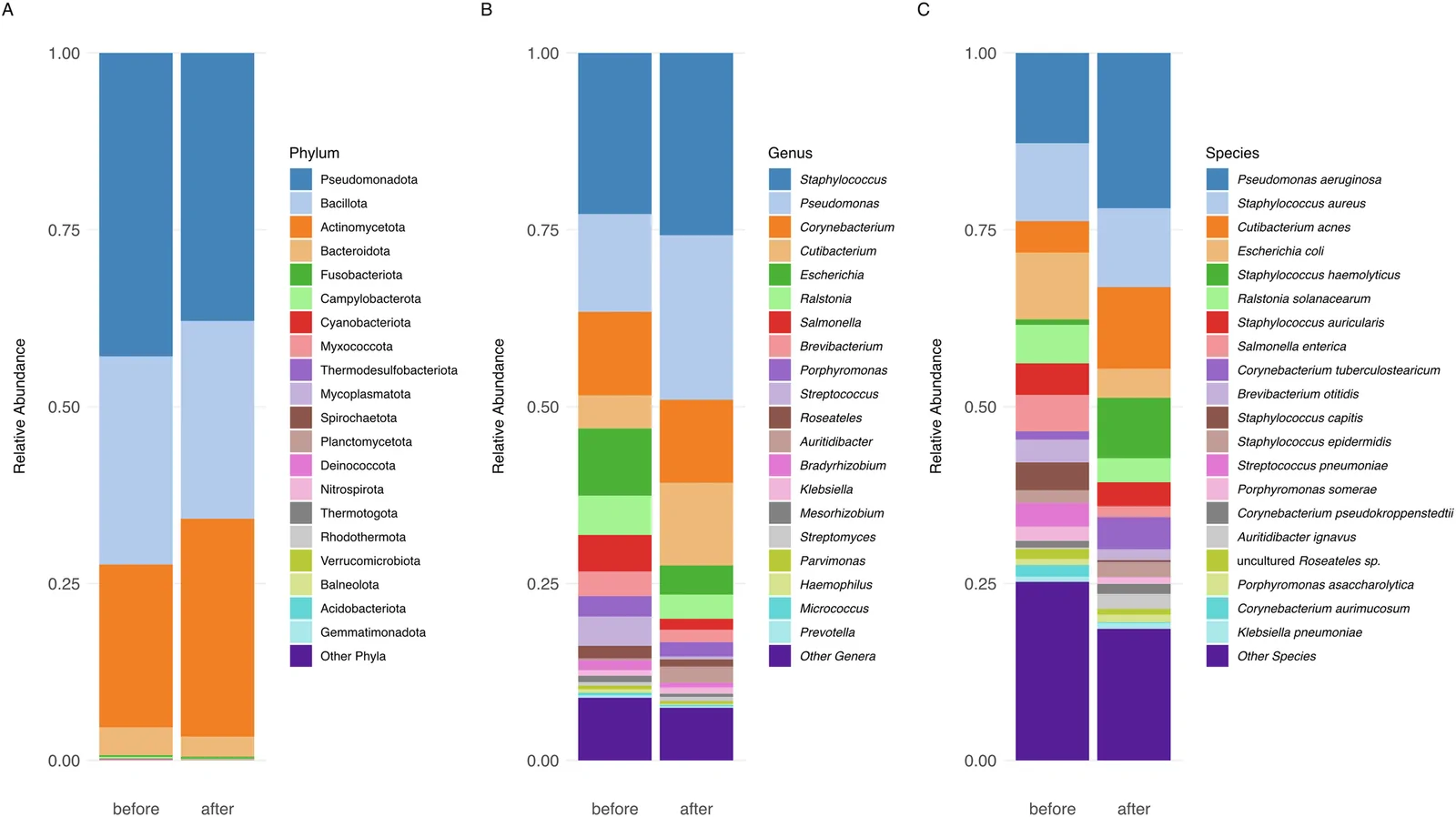
Taxonomic composition of different bacteria. Taxonomic composition of different bacteria (**A**: phylum; **B**: genus; **C**: species of COM patients before and after 14 days of oral 1,8-Cineol administration (3 times per day, 200 mg Soledum forte). Abundances were converted to relative abundances and up to 20 taxa were plotted.

Next, the bacterial and fungal taxonomic composition was analyzed on phylum-, genus-, and species-level before and after 14 days of 1,8-Cineol administration. Data revealed that the identified bacterial communities in COM patients are dominated by Pseudomonadota, Bacillota, and Actinomycetota (Fig. 2).

Differential abundance analysis identified significant changes in 5 different bacterial species (*p* < 0.1), including increased abundances of different *Corynebacterium* species and decreased levels of *Staphylococcus spp*. upon 1,8-Cineol treatment (Fig. 2).

**Fig. 3:**
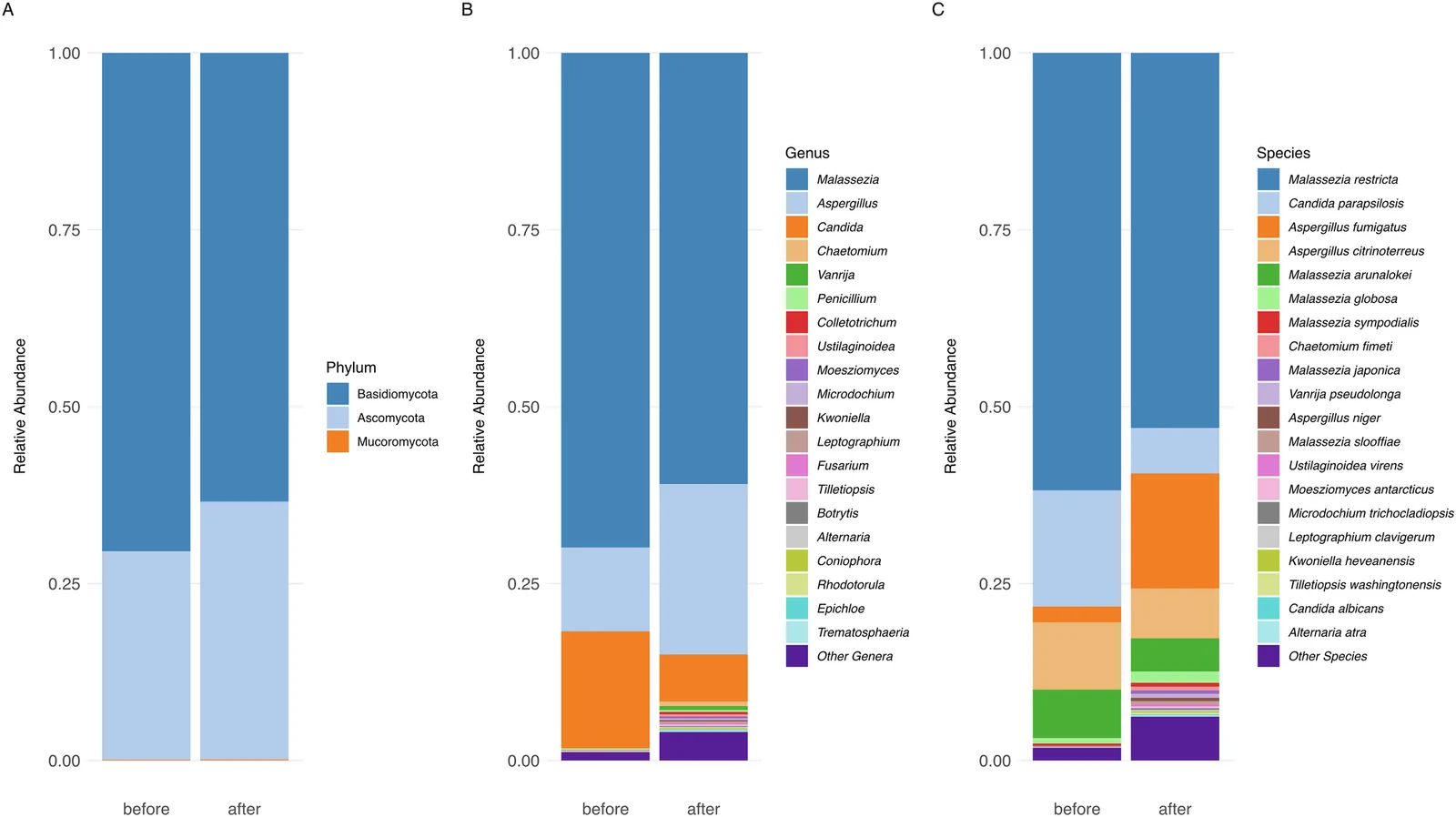
Taxonomic composition of different fungi. Fungal taxonomic composition (**A**: phylum; **B**: genus; **C**: species) of COM patients before and after 14 days of oral 1,8-Cineol administration (3 times per day 200 mg Soledum^®^ Kapseln forte). Abundances were converted to relative abundances and up to 20 taxa were plotted.

Fungal communities were shown to be primarily dominated by Basidiomycota (especially Malassezia) and Ascomycota (Fig. 3).

Significant alterations in 18 distinct fungal species were found by differential abundance analysis (*p* < 0.05).

### Network analysis of microbial communities in response to 1,8-Cineol

Network analysis has emerged as a powerful tool for understanding complex microbial communities by capturing and visualizing the relationships between different taxa.

**Fig. 4:**
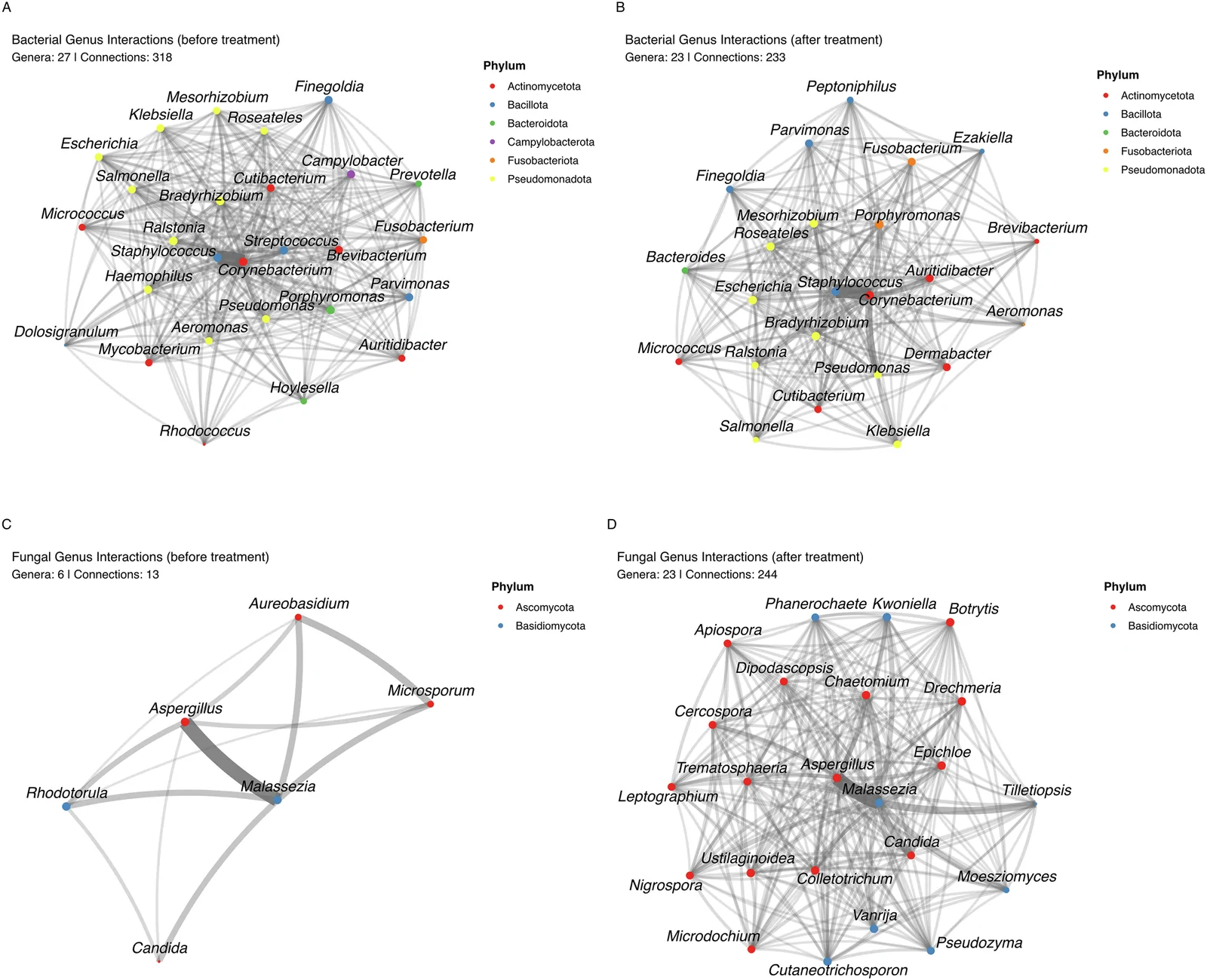
Network analysis of microbial communities in response to 1,8-Cineol. Spearman correlation networks were constructed using species present in at least 10% of the samples with a minimum abundance of 0.1%. Data revealed decreased numbers of bacterial species (from **A**: 64 to **B**: 44), whereas elevated numbers of fungal taxa (from **C**: 10 to D: 33) were observed upon 1,8-Cineol treatment. Interactions were summarized at the genus level for bacteria before (**A**) and after treatment (**B**). Similarly, fungal genus interactions are shown before (**C**) and after treatment (**D**). Line widths denote interaction strength, and node sizes reflect degree centrality (number of interactions).

Spearman correlation networks were constructed using taxa present in at least 10% of the analyzed samples with a minimum abundance of 0.1%. Genus-level interaction networks for bacteria (panels A-B) and fungi (panels C-D) before and after treatment revealed a negative shift of bacterial taxa (from 64 to 44) and elevated numbers of fungal taxa (from 10 to 33) upon 1,8-Cineol treatment (Fig. 4).

The bacterial network before treatment comprised 28 genera with 356 connections, displaying a densely interconnected structure dominated by members of the phyla Actinomycetota, Bacillota, and Pseudomonadota. After treatment, the bacterial network showed modest restructuring to 25 genera with 296 connections, with reduced interaction strengths (thinner edges) and slightly altered connectivity patterns (Fig. 4A, B).

The most striking changes were observed in the fungal networks. Before treatment, the fungal community displayed a sparse interaction network with only 6 genera and 16 connections, primarily involving *Malassezia*, *Candida*, and *Aspergillus* (Fig. 4C).

Following treatment, there was a dramatic expansion to 23 fungal genera forming 257 connections, indicating substantial diversification and increased inter-genus relationships (Fig. 4D). This restructuring was characterized by the emergence of numerous new fungal taxa and a complex web of interactions absent in the pre-treatment community.

However, key bacterial genera maintaining high centrality across both networks included *Staphylococcus*, *Corynebacterium*, and *Pseudomonas*, suggesting their role as core members of the microbial community regardless of treatment status. In the fungal networks, *Malassezia*, *Candida*, and *Aspergillus* maintained prominence but exhibited substantially altered connectivity patterns after treatment, with numerous new connections to emerging fungal genera.

**Fig. 5:**
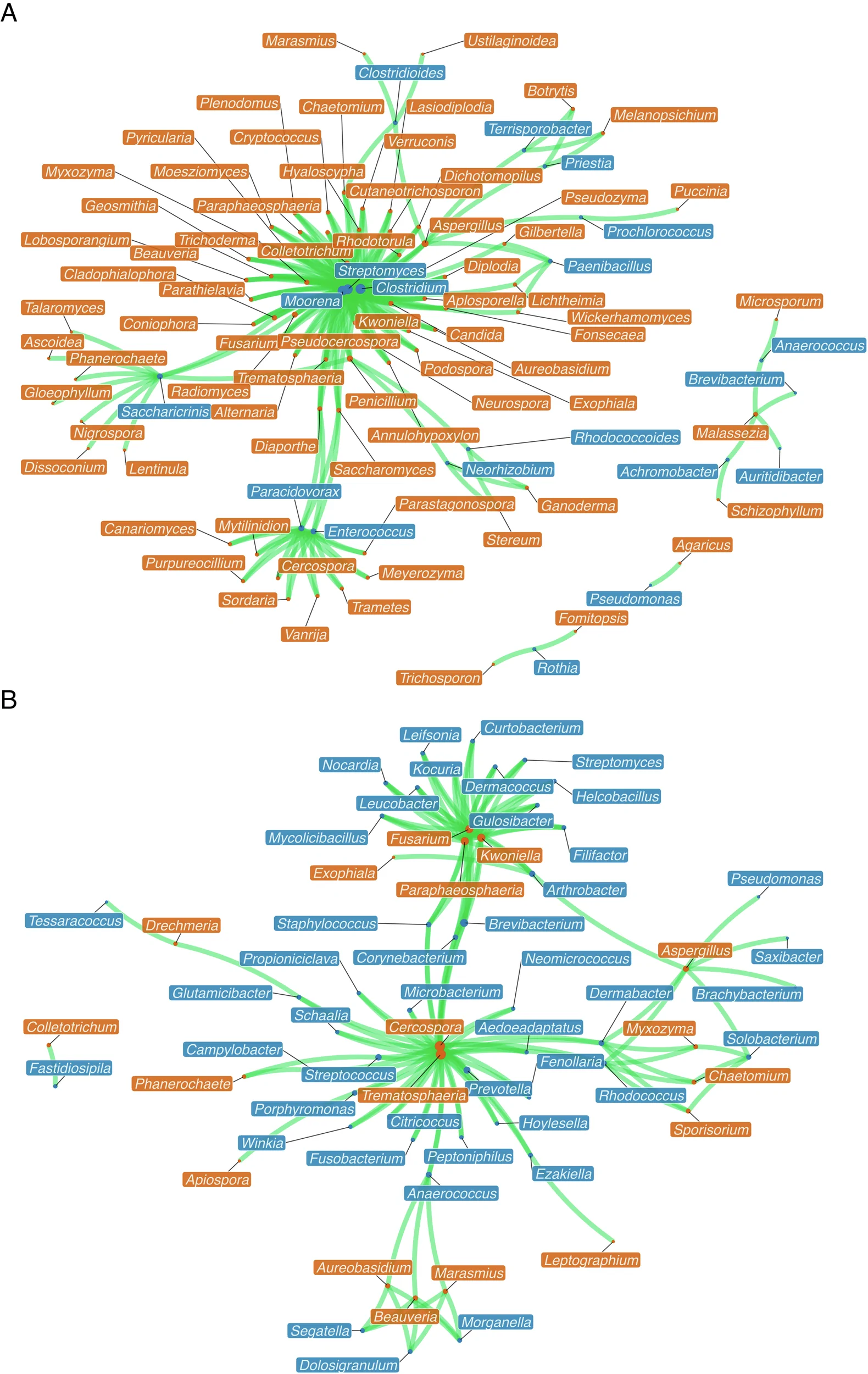
Bacterial and fungal cross-domain network analysis. Cross-domain network analysis of interactions between bacterial and fungal taxa before (**A**) and after **B** 1,8-Cineol treatment. Blue nodes/labels indicate bacteria, and orange nodes/labels represent fungi. Green edges connecting the nodes represent positive correlations between bacterial and fungal genera. The size of nodes corresponds to the number of interactions (degree), while edge thickness indicates the strength of correlation.

Next, cross-domain network analysis was performed, which depicts the potential interactions between bacterial and fungal taxa before and after 1,8-Cineol treatment. The network is organized with nodes representing microbial genera, where blue nodes/labels indicate bacteria and orange nodes/labels represent fungi (Fig. 5). Green edges connecting the nodes represent positive correlations between bacterial and fungal genera. The size of nodes corresponds to the number of interactions (degree), while edge thickness indicates the strength of correlation (Fig. 5).

The pre-treatment situation exhibits extensive cross-domain interactions with numerous fungal taxa (predominantly Ascomycota and Basidiomycota), forming strong connections with a relatively small set of bacterial genera. Key bacterial hub genera include *Pseudomonas*, *Clostridium*, and *Streptomyces*, forming connections with multiple fungal partners. The fungal community shows remarkable diversity, with many genera maintaining cross-domain connections, suggesting a complex, potentially stable community with established bacterial–fungal relationships (Fig. 5A).

The post-treatment network shows a substantial reorganization with fewer total interactions but a more diverse set of bacterial partners. There is a notable shift in bacterial taxa composition, with the emergence of several Actinobacteria and Firmicutes genera (*Corynebacterium*, *Staphylococcus*, *Streptococcus*) forming new cross-domain relationships.

The fungal diversity appears reduced, with fewer fungal genera maintaining strong bacterial connections. The overall network appears more compartmentalized with distinct interaction clusters (Fig. 5B).

Data revealed substantial network changes in response to 1,8-Cineol treatment. Network density increased by 49.9% and indicates a more interconnected bacterial–fungal community, whereas more balanced connectivity between bacteria and fungi was underlined by a decreased domain specialization ratio of 46%. Furthermore, we observed a complete turnover in keystone taxa with almost no overlap in central taxa before versus after treatment. The post treatment situation revealed multiple core node in the fungal community such as *Cercospopra*, *Aspergillus* and *Fusarium* as opposed to *Cladophialophora bantiana* and *Purpureocillium takamizusanense*. Within the bacterial community, a shift from Streptomycetes and Actinomycetes dominance (*Amycolatopsis*, *Actinomyces*) to Proteobacteria/Pseudomonadota (*Serratia*, *Sphingobium*, *Ferribacterium*) could be observed. In contrast, a shift from *Filobasidium floriforme* and *Candida albicans* to *Cladophialophora bantiana* and *Purpureocillium takamizusanense* was identified in the fungal community.

### Microbial communities with regard to therapy response

An evaluation of the clinical course revealed an improved clinical outcome of 13 Otitis media patients before and after 1,8-Cineol treatment from our cohort, who showed no response to the previous standard therapeutic regimen. In addition to the documented improvement in ear symptoms, treatment with 1,8-Cineol also led to an improvement in accompanying rhinosinusitis symptoms.

To assess whether specific signatures of the microbial communities or the described network dynamics would correlate with the improved situation in response to 1,8-Cineol treatment, samples collected before herbal drug administration (T1) were grouped depending on their response status after completing the treatment (T2). Patients presenting with less to no otorrhea and a decrease in pain were defined as responders.

**Fig. 6:**
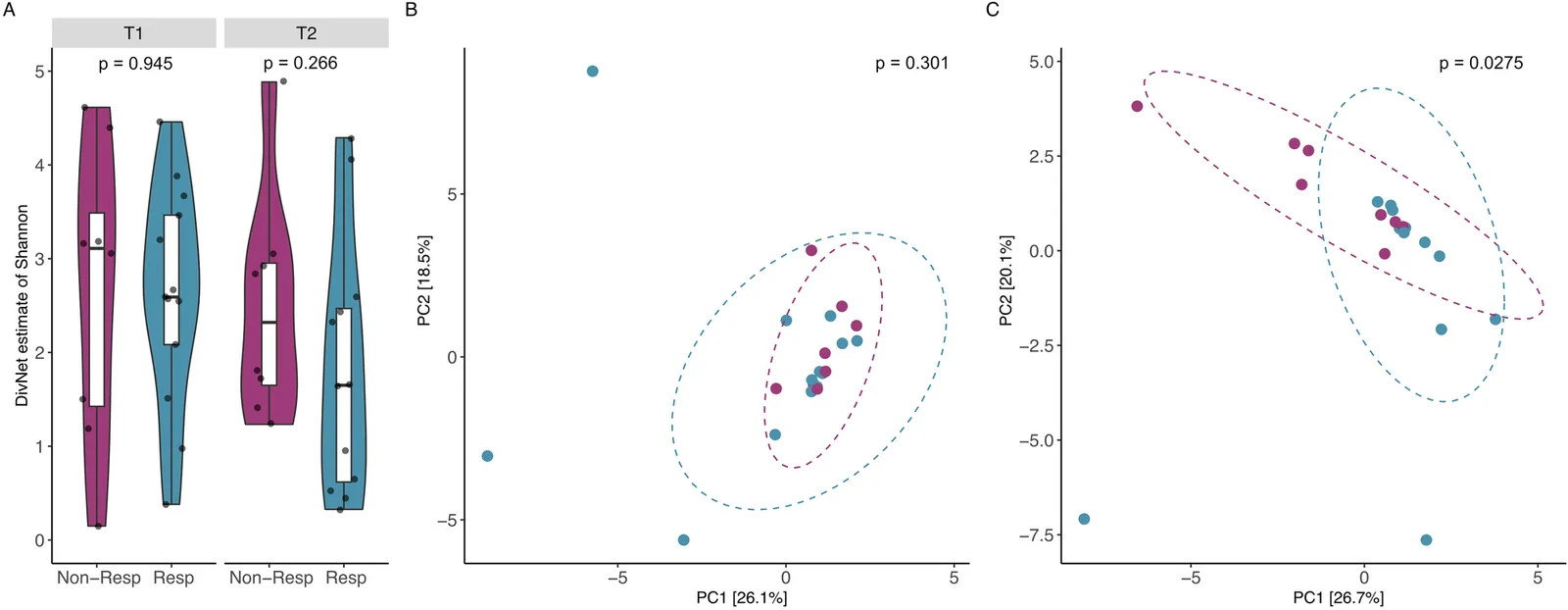
Bacterial alpha and beta diversities. Differences in alpha (**A**) (*p* values derived from betta test) and beta (**B**, **C**) (*p* values derived from PERMANOVA) diversity of bacterial communities in responder and non-responder patient groups before (T1) and after (T2) 1,8-Cineol treatment. Cerulean blue refers to responders and muted magenta to non-responders, respectively.

Analysis of differences in alpha diversity revealed no significant differences concerning the bacterial and fungal communities (Fig. 6A).

Differences in beta diversity were assessed using PERMANOVA and revealed no significant differences for bacteria (T1) and fungi (T1/T2), but significant differences for bacteria after treatment (*p* = 0.0275) (Fig. 6).

**Fig. 7:**
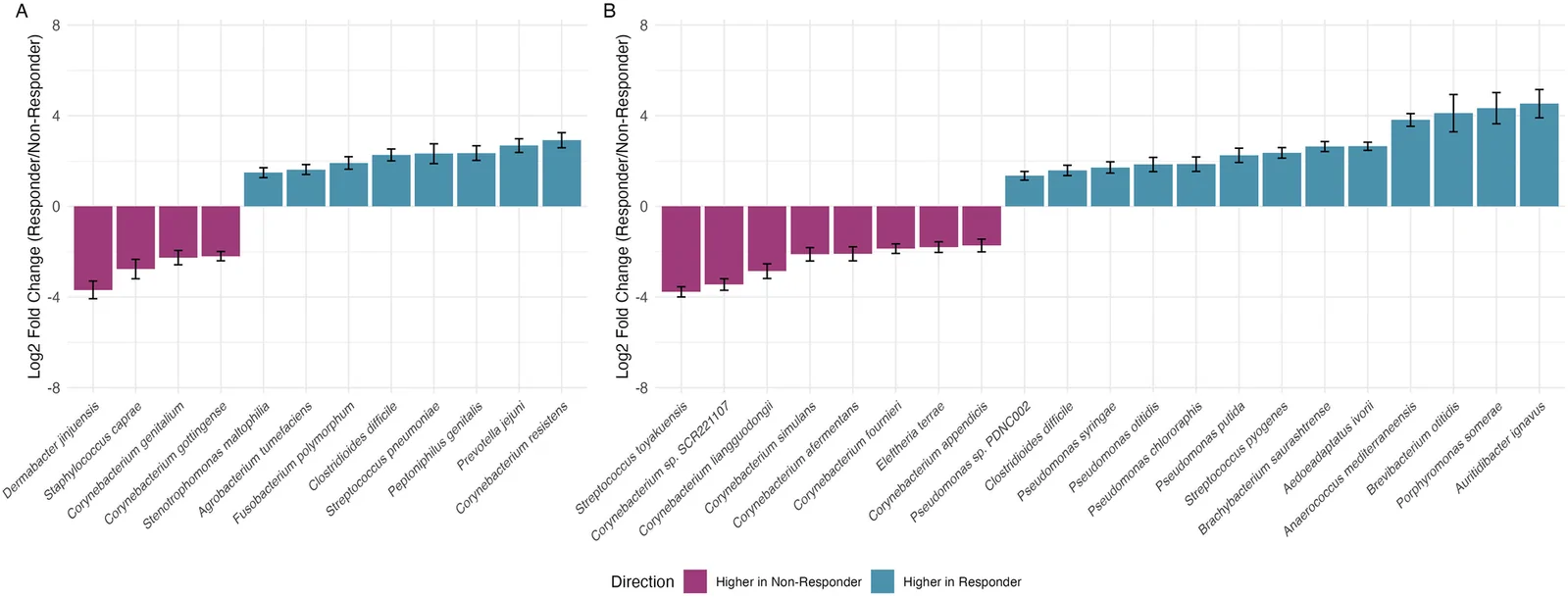
Microbial communities of Cineol responders and non-responders. Comparison of the microbial community composition between responder and non-responder groups revealed significant differences (*p* ˂ 0.001) of different bacterial species before (**A**) and after (**B)** 1,8-Cineol treatment.

Furthermore, a comparison of the microbial community composition between responder and non-responder groups revealed significantly increased abundances (p ˂ 0.001) of different bacterial species in therapy responding patients before 1,8-Cineol treatment, including *Agrobacterium tumefaciens*, *Clostridium difficile*, *Steptococcus pneumoniae, and Dermabacter jinjuensis* (Fig. 7A). In contrast, significantly increased levels of different *Corynebacterium* species (*C. genitalium, C. gottingense, C. resistens*) were identified in the non-responder group (Fig. 7A).

Comparison of the microbial community composition after 14 days of 1,8-Cineol treatment revealed a completely different situation, with more bacterial species at significantly different levels in responders and non-responders. A comparison of baseline clinical characteristics between responders and non-responders revealed no significant differences (see Supplementary Table 1).

In particular, therapy responding COM patients revealed significantly increased abundances of different Gram-negative *Pseudomonas* species (*P. chlororaphis, P. otitidis, P. putida, P. sp. PDNC002, P. syringae*), whereas significantly increased levels of various Gram-positive *Corynebacterium* species (*C. afermentans, C. appemdicis, C. fournieri, C. liangguodongii, C. simulans, C. sp. SCR221107*) were identified in the non-responder group compared to the 1,8-Cineol responding patients (Fig. 7A).

## Discussion

The present study investigated the bacterial and fungal communities and cross-domain network interactions in patients with chronic Otitis media in response to the natural monoterpene 1,8-Cineol as an alternative treatment option.

Earlier studies have already shown anti-microbial effects of 1,8-Cineol in different inflammatory diseases^19–22^. Regarding bacterial colonization, Sokovic et al. examined anti-microbial activities of 1,8-Cineol in Minimal Inhibitory Concentration (MIC) assays and found effects especially against Gram-positive bacteria like *Staphylococcus aureus*. Also, Gram-negative bacteria like *Klebsiella pneumoniae, Proteus vulgaris*, and *Pseudomonas aeruginosa* showed growth reduction in the presence of 1,8-Cineol. They also found stronger anti-microbial capacities of 1,8-Cineol compared to other compounds of the essential eucalyptus oil, like α- and β-Pinene^23^. This effect has already been described in middle ear infections, especially in ears that are colonized by *Pseudomonas aeruginosa*^24,25^.

In the Chinese (Wuhan) study population, clinical otomycosis secretions frequently exhibited *Staphylococcus spp*. as the dominant bacterial genus (average relative abundance approximately 29%) and *Aspergillus spp*. as the dominant fungal genus (average relative abundance approximately 91%). In contrast, in the present study of chronic otitis media (COM) patients from northern Germany, *Pseudomonas aeruginosa* and *Malassezia spp*. predominated^26^.

1,8-Cineol is capable of reducing the biofilm of some bacterial species^27^, and it is most likely that biofilm formation is a major problem concerning the efficacy of anti-microbial treatment due to limited penetrability. This effect of 1,8-Cineol has also been described in otorhinolaryngological cases: Schürmann et al. described the same decrease of biofilm in chronic rhinosinusitis with *Staphylococcus aureus* as the main pathogenic bacteria^22^. Hendry et al. discovered the impact of eucalyptus oil and 1,8-Cineol on the formation of biofilms^28^, and studies on pathogenic *S. aureus* revealed a growth inhibitory effect of 1,8-Cineol but also the downregulation of a protein involved specifically in biofilm formation^22^.

This is a very interesting aspect, since the quorum sensing (QS) pathway is known to be required for the proper formation and functioning of bacterial biofilms. QS is the regulatory communication system via chemical signals within bacterial populations and is involved in bacterial invasion, defense, and distribution^29,30^. Moreover, such analyses of bacterial–fungal interaction networks are particularly relevant in the context of our findings, because they provide a framework to investigate further how dual-species biofilms emerge and behave under in vitro and clinical conditions. Interkingdom signaling through quorum-sensing (QS) molecules and diverse secondary metabolites can modulate growth, morphology, gene expression, and extracellular matrix (ECM) composition, thereby reshaping the spatial organization and architecture of mixed biofilms^31^.

In the present study, network analyses were carried out to identify co-occurrence patterns and dynamics of the bacterial and fungal populations in Otitis media patients, also considering the individual clinical situation and 1,8-Cineol therapy response.

Network analysis provides complementary perspectives on the microbial community. The basic co-occurrence networks characterized overall structural properties, the advanced analysis revealed finer patterns in community organization, and the cross-domain analysis captured inter-kingdom relationships^32^. This is of particular interest in medical research, where the identification of disease-associated microbial key species may help to develop novel therapeutic approaches^33^.

The human body houses an incredible variety of complex microbial communities, including protozoa, viruses, bacteria, and fungi, all of which share the same host and therefore constantly have to compete or cooperate^34–36^.

Until now, primarily bacterial co-occurrence patterns have been studied, while fungal interactions or cross-domain interactions have received far less attention^37,38^. In the present study, the most striking changes were observed in the fungal network upon 1,8-Cineol treatment. While the ear bacteria of COM patients showed modest network restructuring with preserved core genera, the fungal community underwent dramatic diversification and network expansion. This suggests that treatment may have disrupted competitive exclusion mechanisms in the fungal community, allowing for increased diversity and interactions, despite no significant changes in traditional diversity metrics.

Key bacterial genera maintaining high centrality across both networks included *Staphylococcus*, *Corynebacterium*, and *Pseudomonas*, suggesting their role as core members of the microbial community regardless of treatment status. The more balanced cross-domain connectivity may represent a normalization of a previously dysbiotic state and strongly point to potential ecological mechanisms of treatment efficacy beyond simple pathogen reduction.

However, the evaluation of such complex microbial relationships is accompanied by different challenges, since microbial counts represent proportions instead of absolute abundances and thus computational correlation analysis often results in spurious associations between low-abundant microbial^39^. So far, none of the existing tools successfully addresses all issues of microbial network inference, i.e., they are unable to distinguish between competition and cooperation^40^.

Nevertheless, network analysis represents a valuable methodological advancement in microbiome research and allows a comprehensive view of how microbial communities reorganize in response to treatment, in our case, highlighting potential ecological mechanisms underlying therapeutic outcomes in Otitis media patients. Investigations on the microbial community compositions in Otitis media patients upon 1,8-Cineol treatment revealed significantly different microbial signatures in responders and non-responders. In particular, therapy-responding COM patients revealed significantly increased abundances of different gram-negative *Pseudomonas* species, whereas significantly elevated levels of various gram-positive *Corynebacterium* species were observed in the non-responder group compared to the 1,8-Cineol responding patients. In this light, it needs to be considered that both *Corynebacterium* and *Pseudomonas* species are part of a group of bacteria with the potential to cause ear infections^41^. Both share the same niche, which makes certain interactions between those groups of bacteria likely. In fact, a recent report observes *Pseudomonas* biofilm formation being inhibited by a *Corynebacterium* strain^42^. The exact role and contribution of such interaction in the light of COM treatment remains unclear; however, these keystone taxa may represent new targets for therapeutic intervention or biomarkers for treatment response. Given apparent microbial differences between responders and non-responders in 1,8-Cineol treatment in the gut^17^ and (as herein presented) the ear itself, this implies careful consideration of microbial composition and networks in COM when classical treatment regimens fail to improve the disease condition. A limitation of our study is the lack of information on cerumen composition and moisture levels. These parameters may be relevant, as higher incidences of acute otitis externa have been documented in warm, humid environments and during high-humidity seasons^43^. Moreover, cerumen likely contributes to a protective microenvironment through its content of anti-microbial lipids and peptides (e.g., defensins), which exert bacteriostatic effects against many potential otopathogens^44^.

The multimicrobial composition observed in patients with chronic otitis media (COM) following treatment, as well as its modulation in response to 1,8-Cineol administration, holds substantial clinical relevance. This is primarily due to the distinct functional activities of the bacterial and fungal species involved. The detected microorganisms can be categorized into three groups: (i) mutualistic species, which contribute to barrier protection, compete with otopathogens, and support the commensal ear microbiota; (ii) opportunistic microbes, which are typically asymptomatic but may cause infection under immunocompromised conditions; and (iii) primary pathogens, capable of inducing infection even in immunocompetent individuals^45^. In the context of targeted treatment, it is crucial to prevent the transition of commensal or mutualistic bacterial and fungal communities into otopathogenic forms, thereby maintaining a balanced microbial ecosystem within the middle ear and promoting the resolution of otitis rather than the persistence of pathogenic processes.

The microbiome sequencing data presented here are inherently compositional, reflecting relative proportions rather than absolute abundances. This limitation arises from variable DNA extraction efficiency, PCR amplification biases, differences in genome or operon copy numbers, and the normalization of read counts, which collectively remove information on absolute microbial load. Consequently, these data do not permit robust conclusions about absolute bacterial-to-fungal loads before and after treatment^46,47^.

In addition, DNA sequencing alone cannot discriminate between live, dead, and dormant bacterial or fungal cells, nor can it resolve the metabolic state or growth mode of the microorganisms, such as high versus low growth rate, vegetative versus stress responses (for example, oxidative stress or starvation), or biofilm-associated versus planktonic lifestyles^48–50^.

Nevertheless, such analyses of bacterial–fungal interaction networks are particularly relevant in the context of our findings, because they provide a framework to investigate further how dual-species biofilms emerge and behave under in vitro and clinical conditions.

These reciprocal interactions promote physical co-adhesion, ECM sharing, and co-aggregation into mixed microcolonies, which in turn influence biofilm thickness, density, and mechanical stability, e.g., hindering topical drug penetration. Importantly, such network-level reorganization can alter anti-microbial susceptibility profiles, with dual bacterial–fungal biofilms frequently showing increased tolerance or resistance compared with single-species biofilms. However, in some constellations, one partner’s resistance may be reduced. Thus, mapping these networks in COM patients not only describes community composition, but also generates hypotheses on functional consequences such as altered drug responses, persistence of infection, and treatment failure that warrant targeted mechanistic studies of multispecies population dynamics in mixed bacterial–fungal communities^31,51^.

In dual-species biofilms, both *Pseudomonas aeruginosa* and *Candida albicans* exhibit markedly increased biovolume (approximately threefold and sixfold, respectively) compared with their single-species biofilms. This cooperative enhancement is not observed in planktonic co-cultures^52^. In this context, *P. aeruginosa* can simultaneously induce mitochondrial oxidative stress in *C. albicans* and downregulate key fungal detoxification enzymes, thereby preventing *C. albicans* from mounting an adequate oxidative stress response to Amphotericin B and ultimately promoting fungal cell death^53^. Other models highlight strong antibiosis, such as the effect of *Staphylococccus aureus* on *Aspergillus fumigatus* biofilms, which results in scarce biofilm formation, disorganized fungal structures, abortive and limited hyphal growth, and conidia that are reduced in number, display surface alterations and undergo lysis^53^. Likewise, antagonistic and antibiosis relationships between *Aspergillus niger and Staphylococcus aureus* biofilms have been described in otomycosis-related in vitro systems, where *S. aureus* exerts a strong inhibitory effect on *A. niger* biofilm formation and on the expression of biofilm-associated genes^54^.

Besides the temporal changes observed during the 14‑day course of 1,8-Cineol treatment, we cannot exclude the possibility that secondary bacterial colonization or additional viral influence, originating from the external auditory canal or nasopharyngeal sources, contributed to the observed recolonization^55,56^. Furthermore, no multiple testing correction was applied to the differential abundance analysis and therefore results should be interpreted as hypothesis-generating.

In conclusion, these data indicate that the mucosal microbiomes in chronic and complicated ear infections consist of polymicrobial communities, in which individual microbial populations respond differently to treatment. The resulting shifts in these complex, multi-pathogen–driven inflammatory environments may lead to substantial remodeling of bacterial–fungal network interactions.

## Methods

### Ethics Statement

Medical examinations and surgical treatments were carried out at the Department of Otorhinolaryngology, University Hospital Schleswig-Holstein, Campus Luebeck. All patients have given their written informed consent. The observational study was approved by the local ethics committee of the University of Luebeck (approval number 22-040) and conducted in accordance with the ethical principles for medical research formulated in the WMA Declaration of Helsinki.

### Characteristics of examined patients

All enrolled patients (*n* = 22) in this study suffered from persistent COM and rhinosinusitis, both of which share the same respiratory epithelium. The mean age of the patients with COM was 53 years (30 to 68 years), and all patients underwent antibiotic therapy and middle ear surgery, and developed microbial resistance against many or all available classical antibiotics. Often, there were no therapeutic options left, and therefore, we treated them with 1,8-Cineol. 1,8-Cineol (CNL-1976) was used in terms of the clinically approved drug Soledum^®^ Kapseln forte (capsules) (Cassella-med GmbH & Co. KG, Cologne, Germany). For therapeutic use, patients have been prescribed Soledum capsules forte (3 × 200 mg Cineol/day) over 14 days for oral administration. None of the patients experienced any adverse events in response to 1,8-Cineol treatment. They did not obtain any local or systemic antibiotic treatments during this time period.

To participate in the study, patients must present with therapy-resistant chronic otitis media that has failed to improve despite both local and systemic antibiotic treatment. In addition, eligible patients must exhibit persistent otorrhea lasting longer than 14 days or have experienced more than three episodes of ear discharge within the previous year. Patients diagnosed with cholesteatoma are explicitly excluded from participation in the study.

For diagnostic scoring, we examined the change of pain, hearing loss, otorrhea, pressure in the tuba auditive, and other factors related to quality of life with a questionnaire before and after off-label therapy with 1,8-Cineol administration. Patients received an information sheet containing detailed instructions and safety information for the intake of the 1,8-cineole tablets. The sheet included a table that allowed patients to document and monitor their medication intake by checking off each dose taken. In addition, patients could record any side effects or other relevant observations. This form was used to assess and monitor patient adherence throughout the study. The following criteria were defined to identify responders to the treatment: (I) Complete dryness of the external auditory canal; (II) Significant reduction in otorrhea with absence of fluid discharge; (III) Reduction of at least 3 points on the Visual Analog Scale (VAS) for pain.

### Analysis of ear swabs for microbial colonization

Ear swab samples were collected from the auditory canal of the COM patients by insertion of a sterile flexible cotton-wool swab (Copan eNat Swab; Mast Diagnostik GmbH, Reinfeld, Germany). Swabs were stored in sterile Skim-Milk-Tryptone-Glucose-Glycerol-Broth and later used for DNA isolation and analysis of the bacterial and fungal communities. The DNA was extracted using the DNeasy Power Soil Pro Kit (Qiagen GmbH, Hilden, Germany) according to the protocol. For homogenization, the samples were pretreated in a Precellys 24 at 5000 rpm for 2 × 20 s. The extracted DNA was fragmented with purified Tn5 transposase expressed in-house, according to the protocol by Picelli et al.^57^. Each sample was individually barcoded (single-indexed as N5XX with N7XX variable barcodes; the oligonucleotides were synthesized by metabion international AG, Planegg, Germany) and pooled equimolarly for high-throughput sequencing by a NextSeq 500 (Illumina, San Diego, California, USA). Prepared libraries were sequenced with a NextSeq 500/550 Mid Output Kit v2.5 using 2x150bp cycles on an Illumina NextSeq 500.

### Sequencing data processing and analysis

Raw sequencing reads were processed using the nf-core/detaxizer pipeline (v1.1.0), a Nextflow-based workflow for quality control, preprocessing, and taxonomic classification of metagenomic samples^58^. Briefly, quality control and adapter trimming were performed using BBDuk from the BBTools suite (v39.1). Host contamination was removed by mapping reads against the human reference genome (GRCh38) using BBMap. Taxonomic classification of decontaminated reads was conducted using Kraken2 (v2.1.3) with the standard database^59^. Post-classification filtering was applied to remove false-positive taxonomic assignments. The pipeline was executed using Singularity containers (v3.8.0) [6] to ensure computational reproducibility. Downstream analysis sample sheets compatible with nf-core/taxprofiler [7] and nf-core/mag [8] were automatically generated for subsequent analyses. Downstream analysis sample sheets compatible with nf-core/taxprofiler were automatically generated for subsequent analyses. Subsequently, processed reads were profiled using the nf-core/taxprofiler pipeline (v1.2.3) employing three complementary taxonomic classifiers: Kraken2 (v2.1.3) operating in quick mode for rapid k-mer-based classification, Bracken (v2.9) for Bayesian abundance re-estimation at the species level with 150 bp read length parameter, and Kaiju (v1.10.0) for protein-level classification using the NCBI nr database^60,61^. The pipeline was configured to skip preprocessing quality control steps as reads had already been processed through the detaxizer workflow. Results were evaluated, and Bracken classifications were used for downstream analysis.

Alpha diversity (Shannon diversity) was estimated using DivNet (v0.4.0), a method that accounts for compositional structure and network effects in microbial communities^62^. Statistical comparisons of alpha diversity between treatment groups were performed using the betta function from breakaway (v4.8.4), which appropriately incorporates variance estimates from diversity calculations^63^. For responder analysis, comparisons were stratified by timepoint (T1: before treatment; T2: after treatment) to assess whether baseline diversity predicted treatment response.

Beta diversity was assessed using Aitchison distance, the appropriate metric for compositional data^39^. Microbiome count data were transformed using centered log-ratio (CLR) transformation via the microbiome package (v1.32.0). Ordination was performed using Redundancy Analysis (RDA) with the vegan package (v2.7-2). Statistical significance of community composition differences between groups was tested using permutational multivariate analysis of variance (PERMANOVA) implemented through the adonis2 function with 9999 permutations. For responder analyses, PERMANOVA was performed separately at each timepoint (T1 and T2) to identify timepoint-specific differences in microbial community structure between responders and non-responders.

Differentially abundant taxa between treatment groups were identified using ANCOM-BC2 (ANCOMBC v2.12.0), which addresses the compositionality of microbiome data through bias correction^64^. Analyses were performed at the species level with treatment status (or responder status) as the fixed effect, and no random effects were specified. Taxa were retained if they passed the ANCOM-BC2 structural sensitivity analysis (passed_ss = TRUE) and showed a nominal *p* value < 0.05 (bacteria) or <0.1 (fungi, reflecting lower statistical power due to reduced sample size after filtering), combined with an absolute log2 fold change >0.5. No additional multiple testing correction was applied given the exploratory nature of the analysis and the small sample size, which precluded meaningful FDR estimation. For the responder analysis, differential abundance testing was conducted separately at baseline (T1) and post-treatment (T2) to identify taxa associated with treatment response at each timepoint.

Furthermore, we employed a three-tiered approach to network analysis, progressively increasing in complexity and specificity, to comprehensively characterize the bacterial and fungal communities in otitis media before and after 1,8-Cineol treatment.

Our network analysis framework consisted of three distinct but complementary approaches:

### Basic co-occurrence network analysis

The initial analysis established foundational co-occurrence networks separately for bacterial and fungal communities. Due to computational constraints with more complex methods like SpiecEasi, we employed Spearman correlation to detect significant associations between taxa. This correlation-based approach allowed us to identify significant co-occurrence patterns within each domain (bacteria-bacteria and fungi-fungi) and characterize network properties, including density, modularity, and centrality measures. By comparing network metrics before and after treatment, we could assess overall structural changes within each microbial community independently.

### Advanced network visualization and analysis

Building upon these foundational networks, our second approach enhanced the visualization and analytical depth by focusing on module structures, taxonomic composition of modules, and genus-level interactions. This analysis revealed more nuanced patterns in community organization by examining how closely related taxa formed coherent modules and how these modules changed following treatment. Additionally, we examined network resilience and analyzed shifts in hub taxa, which represent potential keystone species that may disproportionately influence community structure and function.

### Cross-domain network analysis

The most sophisticated component of our analytical framework transcended individual domains to explore interactions between bacterial and fungal communities. This approach enabled us to investigate inter-kingdom relationships that may play critical roles in microbiome dynamics but would be missed by single-domain analyses. We constructed bipartite networks connecting bacterial and fungal taxa, calculated specialized bipartite network metrics including domain assortative and specialization ratios, and identified cross-domain keystone taxa. This cross-domain perspective allowed us to assess whether treatment-induced changes in community structure were coordinated across kingdoms, potentially revealing mechanisms of interaction between bacteria and fungi in the pathogenesis or resolution of otitis media.

All statistical analyses and figure generation were performed in R. The code used to produce the figures presented in this study is publicly available at https://www.ebi.ac.uk/ena/browser/view/PRJEB104438.

## Supplementary information

Supplementary Table 1 REVISION.

## Data Availability

The data presented in this study are available on request from the corresponding author. Raw sequences used for microbiome analysis within this study are made available at the European Genome-phenome Archive (EGA) under accession number PRJEB104438. Code Availability Statement: The R code used for all statistical analyses and figure generation in this study is openly available at https://www.ebi.ac.uk/ena/browser/view/PRJEB104438.

## References

[1] Monasta, L. et al. Burden of disease caused by otitis media: Systematic review and global estimates. *PloS One* **7**, e36226 (2012). 22558393 10.1371/journal.pone.0036226 PMC3340347

[2] Perez-Herrera, L. C. et al. Associated factors, health-related quality of life, and reported costs of chronic otitis media in adults at two otologic referral centers in a middle-income country. *PloS One* **15**, e0244797 (2020). 33382816 10.1371/journal.pone.0244797 PMC7775072

[3] Rovers, M. M., Schilder, A. G., Zielhuis, G. A. & Rosenfeld, R. M. Otitis media. *Lancet* **363**, 465–473 (2004). 14962529 10.1016/S0140-6736(04)15495-0

[4] Bruchhage, K. L. et al. Hearing rehabilitation and microbial shift after middle ear surgery with Vibrant Soundbridge in patients with chronic otitis media. *Eur. Arch. Otorhinolaryngol.* **280**, 3107–3118 (2023). 36662266 10.1007/s00405-022-07795-9 PMC10220096

[5] Leichtle, A., Hoffmann, T. K. & Wigand, M. C. [Otitis media: Definition, pathogenesis, clinical presentation, diagnosis and therapy]. *Laryngorhinootologie* **97**, 497–508, (2018). 10.1055/s-0044-101327 29986368

[6] Jiang, H. et al. Bacterial and fungal infections promote the bone erosion progression in acquired cholesteatoma revealed by metagenomic next-generation sequencing. *Front Microbiol* **12**, 761111 (2021). 34803987 10.3389/fmicb.2021.761111 PMC8604023

[7] Kazmierczak, W. et al. Analysis of pathogens and antimicrobial treatment in different groups of patients with chronic otitis media. *J. Laryngol. Otol.* **136**, 219–222 (2022). 34702380 10.1017/S0022215121003224

[8] Chai, Y. et al. Intratympanic lipopolysaccharide elevates systemic fluorescent gentamicin uptake in the cochlea. *Laryngoscope* **131**, E2573–E2582 (2021). 33956344 10.1002/lary.29610 PMC8453712

[9] Khatun, M. R., Alam, K. M. F., Naznin, M. & Salam, M. A. Microbiology of chronic suppurative otitis media: An update from a tertiary care hospital in Bangladesh. *Pak. J. Med. Sci.* **37**, 821–826 (2021). 34104172 10.12669/pjms.37.3.3942 PMC8155403

[10] Forge, A. & Schacht, J. Aminoglycoside antibiotics. *Audio Neurootol.* **5**, 3–22 (2000). 10.1159/000013861 10686428

[11] Dai, C. F., Mangiardi, D., Cotanche, D. A. & Steyger, P. S. Uptake of fluorescent gentamicin by vertebrate sensory cells in vivo. *Hear Res.* **213**, 64–78 (2006). 16466873 10.1016/j.heares.2005.11.011 PMC2424187

[12] Di Pietro, P. et al. Monitoring adherence to guidelines of antibiotic use in pediatric pneumonia: the MAREA study. *Ital. J. Pediatr.* **43**, 113 (2017). 29273072 10.1186/s13052-017-0432-2 PMC5741879

[13] Pries, R., Jeschke, S., Leichtle, A. & Bruchhage, K. L. Modes of action of 1,8-cineol in infections and inflammation. *Metabolites* **13**, 751–762 (2023). 37367909 10.3390/metabo13060751 PMC10301542

[14] Juergens, U. R. Anti-inflammatory properties of the monoterpene 1.8-cineole: Current evidence for co-medication in inflammatory airway diseases. *Drug Res. (Stuttg.)* **64**, 638–646 (2014). 24831245 10.1055/s-0034-1372609

[15] MacKenzie, C. et al. Determination of orally administered 1,8-Cineol in nasal polyp tissues from chronic rhinosinusitis patients using gas chromatography: Mass spectrometry. *Sci. Rep.* **13**, 3605 (2023). 10.1038/s41598-023-29941-x PMC9984394 36869061

[16] Graspeuntner, S. et al. Gut microbial communities in chronic rhinosinusitis patients in response to 1,8-Cineol treatment. *Curr. Res. Micro Sci.* **9**, 100442 (2025). 10.1016/j.crmicr.2025.100442 PMC12332871 40785792

[17] Leichtle, A. et al. Anti-inflammatory response to 1,8-Cineol and associated microbial communities in Otitis media patients. *Sci. Rep.* **14**, 16362 (2024). 39014066 10.1038/s41598-024-67498-5 PMC11252366

[18] Rowan-Nash, A. D., Korry, B. J., Mylonakis, E. & Belenky, P. Cross-domain and viral interactions in the microbiome. *Microbiol. Mol. Biol. Rev.* **83**, e00044-–18 (2019). 30626617 10.1128/MMBR.00044-18 PMC6383444

[19] Moo, C. L. et al. Antimicrobial activity and mode of action of 1,8-cineol against carbapenemase-producing Klebsiella pneumoniae. *Sci. Rep.* **11**, 20824 (2021). 34675255 10.1038/s41598-021-00249-y PMC8531306

[20] Juergens, U. R. et al. Anti-inflammatory activity of 1.8-cineol (eucalyptol) in bronchial asthma: a double-blind placebo-controlled trial. *Respir. Med.* **97**, 250–256 (2003). 12645832 10.1053/rmed.2003.1432

[21] Bruchhage, K. L. et al. 1,8-cineol inhibits the Wnt/beta-catenin signaling pathway through GSK-3 dephosphorylation in nasal polyps of chronic rhinosinusitis patients. *Eur. J. Pharm.* **835**, 140–146 (2018). 10.1016/j.ejphar.2018.07.060 30081034

[22] Schurmann, M. et al. The therapeutic effect of 1,8-cineol on pathogenic bacteria species present in chronic rhinosinusitis. *Front Microbiol.* **10**, 2325 (2019). 31708879 10.3389/fmicb.2019.02325 PMC6821979

[23] Sokovic, M., Glamoclija, J., Marin, P. D., Brkic, D. & van Griensven, L. J. Antibacterial effects of the essential oils of commonly consumed medicinal herbs using an in vitro model. *Molecules* **15**, 7532–7546 (2010). 21030907 10.3390/molecules15117532 PMC6259430

[24] Daniel, M., Imtiaz-Umer, S., Fergie, N., Birchall, J. P. & Bayston, R. Bacterial involvement in otitis media with effusion. *Int. J. Pediatr. Otorhinolaryngol.* **76**, 1416–1422 (2012). 22819485 10.1016/j.ijporl.2012.06.013

[25] Hall-Stoodley, L. & Stoodley, P. Evolving concepts in biofilm infections. *Cell Microbiol.* **11**, 1034–1043 (2009). 19374653 10.1111/j.1462-5822.2009.01323.x

[26] Chen, Z. & Zhao, Z. Study on the microbial diversity of ear canal secretions from patients with otomycosis. *Front. Surg.* **11**, 1277799 (2024). 38450054 10.3389/fsurg.2024.1277799 PMC10916698

[27] Merghni, A. et al. Assessment of the antibiofilm and antiquorum sensing activities of Eucalyptus globulus essential oil and its main component 1,8-cineole against methicillin-resistant Staphylococcus aureus strains. *Micro Pathog.* **118**, 74–80 (2018). 10.1016/j.micpath.2018.03.006 29522803

[28] Hendry, E. R., Worthington, T., Conway, B. R. & Lambert, P. A. Antimicrobial efficacy of eucalyptus oil and 1,8-cineole alone and in combination with chlorhexidine digluconate against microorganisms grown in planktonic and biofilm cultures. *J. Antimicrobial Chemother.* **64**, 1219–1225 (2009). 10.1093/jac/dkp362 19837714

[29] Annapoorani, A., Jabbar, A. K., Musthafa, S. K., Pandian, S. K. & Ravi, A. V. Inhibition of quorum sensing mediated virulence factors production in urinary pathogen serratia marcescens PS1 by marine sponges. *Indian J. Microbiol.* **52**, 160–166 (2012). 23729876 10.1007/s12088-012-0272-0 PMC3386442

[30] LaSarre, B. & Federle, M. J. Exploiting quorum sensing to confuse bacterial pathogens. *Microbiol. Mol. Biol. Rev.* **77**, 73–111 (2013). 23471618 10.1128/MMBR.00046-12 PMC3591984

[31] Ruiz, A. et al. The architecture of a mixed fungal-bacterial biofilm is modulated by quorum-sensing signals. *Environ. Microbiol.* **23**, 2433–2447 (2021). 33615654 10.1111/1462-2920.15444

[32] Matchado, M. S. et al. Network analysis methods for studying microbial communities: A mini review. *Comput Struct. Biotechnol. J.* **19**, 2687–2698 (2021). 34093985 10.1016/j.csbj.2021.05.001 PMC8131268

[33] Petrosino, J. F. The microbiome in precision medicine: The way forward. *Genome. Med.* **10**, 12 (2018). 29471863 10.1186/s13073-018-0525-6 PMC5824491

[34] Salazar, N. & de Los Reyes-Gavilan, C. G. Editorial: Insights into microbe-microbe interactions in human microbial ecosystems: Strategies to be competitive. *Front Microbiol.* **7**, 1508 (2016). 27721810 10.3389/fmicb.2016.01508 PMC5033985

[35] Kamada, N., Seo, S. U., Chen, G. Y. & Nunez, G. Role of the gut microbiota in immunity and inflammatory disease. *Nat. Rev. Immunol.* **13**, 321–335 (2013). 23618829 10.1038/nri3430

[36] Huseyin, C. E., O’Toole, P. W., Cotter, P. D. & Scanlan, P. D. Forgotten fungi-the gut mycobiome in human health and disease. *FEMS Microbiol. Rev.* **41**, 479–511 (2017). 28430946 10.1093/femsre/fuw047

[37] Ghannoum, M. A. et al. Characterization of the oral fungal microbiome (mycobiome) in healthy individuals. *PLoS Pathog.* **6**, e1000713 (2010). 20072605 10.1371/journal.ppat.1000713 PMC2795202

[38] Sogin, M. L. et al. Microbial diversity in the deep sea and the underexplored “rare biosphere. *Proc. Natl. Acad. Sci. USA.* **103**, 12115–12120 (2006). 16880384 10.1073/pnas.0605127103 PMC1524930

[39] Gloor, G. B., Macklaim, J. M., Pawlowsky-Glahn, V. & Egozcue, J. J. Microbiome datasets are compositional: And this is not optional. *Front Microbiol.* **8**, 2224 (2017). 29187837 10.3389/fmicb.2017.02224 PMC5695134

[40] Faust, K. & Raes, J. Microbial interactions: From networks to models. *Nat. Rev. Microbiol.* **10**, 538–550 (2012). 22796884 10.1038/nrmicro2832

[41] Mitchell, B. I. & Markantonis, J. E. An underestimated pathogen: Corynebacterium species. *J. Clin. Microbiol.* **63**, e0155224 (2025). 40833082 10.1128/jcm.01552-24 PMC12506004

[42] Ortiz Moyano, R. et al. Antagonistic effects of corynebacterium pseudodiphtheriticum 090104 on respiratory pathogens. *Microorganisms* **12**, 1295 (2024). 39065064 10.3390/microorganisms12071295 PMC11278748

[43] Mittal, A. & Kumar, S. Role of pH of external auditory canal in acute otitis externa. *Indian J Otolaryngol Head Neck Surg*. **66**, 86–91 (2014). 10.1007/s12070-013-0684-0 PMC3938709 24605308

[44] Paprocka, P. et al. The Importance of Ear Canal Microbiota and Earwax in the Prevention of Outer Ear Infections. *Int. J. Mol. Sci.* **27**, 622 (2026). 41596275 10.3390/ijms27020622 PMC12841003

[45] Burton, M. et al. The adult microbiome of healthy and otitis patients: Definition of the core healthy and diseased ear microbiomes. *PloS one* **17**, e0262806 (2022). 35073343 10.1371/journal.pone.0262806 PMC8786117

[46] Kumar, M. S. et al. Analysis and correction of compositional bias in sparse sequencing count data. *BMC Genomics* **19**, 799 (2018). 30400812 10.1186/s12864-018-5160-5 PMC6219007

[47] Boshuizen, H. C. & Te Beest, D. E. Pitfalls in the statistical analysis of microbiome amplicon sequencing data. *Mol. Ecol. Resour.* **23**, 539–548 (2023). 36330663 10.1111/1755-0998.13730

[48] Wang, Y. et al. RNA-based amplicon sequencing is ineffective in measuring metabolic activity in environmental microbial communities. *Microbiome* **11**, 131 (2023). 37312147 10.1186/s40168-022-01449-y PMC10262425

[49] Deng, H., He, C., Worden, A. Z. & Gong, J. Employing a triple metabarcoding approach to differentiate active, dormant and dead microeukaryotes in sediments. *Environ. Microbiol.* **26**, e16615 (2024). 38501240 10.1111/1462-2920.16615

[50] Heidrich, V. & Beule, L. Are short-read amplicons suitable for the prediction of microbiome functional potential? A critical perspective. *iMeta* **1**, e38 (2022). 38868716 10.1002/imt2.38 PMC10989910

[51] Dittmer, M. et al. Quantitative insights and visualization of antimicrobial tolerance in mixed-species biofilms. *Biomedicines* **11**, 2640 (2023). 37893014 10.3390/biomedicines11102640 PMC10604264

[52] Kasetty, S., Mould, D. L., Hogan, D. A. & Nadell, C. D. Both pseudomonas aeruginosa and Candida albicans accumulate greater biomass in dual-species biofilms under flow. *mSphere* **6**, e0041621 (2021). 34160236 10.1128/mSphere.00416-21 PMC8265656

[53] Alam, F. et al. Pseudomonas aeruginosa increases the susceptibility of Candida albicans to amphotericin B in dual-species biofilms. *J. Antimicrobial Chemother.* **78**, 2228–2241 (2023). 10.1093/jac/dkad228 PMC10477122 37522316

[54] Al-Oebady, M. A. H. Study of the antagonistic relationship between gene expression biofilm of Aspergillus niger and Staphylococcus aureus that cause otomycosis. *Curr. Med. Mycol.* **10**, e2024 (2024). 10.22034/cmm.2024.345248.1586 PMC12257041 40662148

[55] Marom, T., Nokso-Koivisto, J. & Chonmaitree, T. Viral-bacterial interactions in acute otitis media. *Curr. allergy asthma Rep.* **12**, 551–558 (2012). 22968233 10.1007/s11882-012-0303-2 PMC3493715

[56] Jotic, A. et al. Biofilm Formation and Its Relationship with the Microbiome in Pediatric Otitis Media. *Microorganisms* **13**, 2760 (2025). 41471964 10.3390/microorganisms13122760 PMC12735705

[57] Picelli, S. et al. Tn5 transposase and tagmentation procedures for massively scaled sequencing projects. *Genome Res.* **24**, 2033–2040 (2014). 25079858 10.1101/gr.177881.114 PMC4248319

[58] Di Tommaso, P. et al. Nextflow enables reproducible computational workflows. *Nat. Biotechnol.* **35**, 316–319 (2017). 28398311 10.1038/nbt.3820

[59] Wood, D. E., Lu, J. & Langmead, B. Improved metagenomic analysis with Kraken 2. *Genome Biol.* **20**, 257 (2019). 31779668 10.1186/s13059-019-1891-0 PMC6883579

[60] Ewels, P. A. et al. The nf-core framework for community-curated bioinformatics pipelines. *Nat. Biotechnol.* **38**, 276–278 (2020). 32055031 10.1038/s41587-020-0439-x

[61] Lu, J., Breitwieser, F. P., Thielen, P. & Salzberg, S. L. Bracken: estimating species abundance in metagenomics data. *PeerJ Comput Sci.* **3**, e104 (2017). 40271438 10.7717/peerj-cs.104 PMC12016282

[62] Willis, A. D. & Martin, B. D. Estimating diversity in networked ecological communities. *Biostatistics* **23**, 207–222 (2022). 32432696 10.1093/biostatistics/kxaa015 PMC8759443

[63] Willis, A. & Bunge, J. Estimating diversity via frequency ratios. *Biometrics* **71**, 1042–1049 (2015). 26038228 10.1111/biom.12332

[64] Lin, H. & Peddada, S. D. Multigroup analysis of compositions of microbiomes with covariate adjustments and repeated measures. *Nat. Methods* **21**, 83–91 (2024). 38158428 10.1038/s41592-023-02092-7 PMC10776411

