# Supplementary material for: Bacterial and fungal communities and network dynamics in Otitis media patients upon 1,8-Cineol treatment
Source: NPJ Syst Biol Appl. 2026 Aug 11;12:135. doi: 10.1038/s41540-026-00808-x (PMC13570884; doi:10.1038/s41540-026-00808-x)
Supplement: Supplementary file 1 [file 41540_2026_808_MOESM1_ESM.docx]

**Supplementary Table 1.**

Comparison of baseline clinical characteristics between responders and non-responders.

| n=22 | Responder  n=14 (63%) | Non-Responder  n=8 (36%) | p-Value |
| --- | --- | --- | --- |
| Age (average) | 55y | 54y | p = 0,956 |
| Sex | women: n=10 (71%)  men: n=4 (29%) | women: n=3 (37,5%)  men: n=5 (62,5%) | p = 0,19  p = 0,17 |
| Diabetes mellitus | n=2 (14%) | n=2 (25%) | p = 1,000 |
| Rheumatic condition | n=2 (14%) | n=1 (12,5%) | p = 1,000 |
| Tympanoplasty | n=10 (71%) | n=8 (100%) | p = 0,12 |
| Tympanic drainage | n=4 (29%) | n=0 (0%) | p = 0,12 |
| Chronic mesotympanic otitis media | n=7 (50%) | n=5 (62,5%) | p = 0,67 |
| Chronic granulating otitis media | n=3 (21%) | n=3 (37,5%) | p = 0,63 |
| Chronic otitis with inner ear involvement | n=4 (29%) | n=0 (0%) | p = 0,12 |
|  |  |  |  |

# For continuous variables such as age, a t-test was used and the other parameters Fisher's exact test was used due to the small sample size.
